# The Reality of Pervasive Transcription

**DOI:** 10.1371/journal.pbio.1000625

**Published:** 2011-07-12

**Authors:** Michael B. Clark, Paulo P. Amaral, Felix J. Schlesinger, Marcel E. Dinger, Ryan J. Taft, John L. Rinn, Chris P. Ponting, Peter F. Stadler, Kevin V. Morris, Antonin Morillon, Joel S. Rozowsky, Mark B. Gerstein, Claes Wahlestedt, Yoshihide Hayashizaki, Piero Carninci, Thomas R. Gingeras, John S. Mattick

**Affiliations:** 1 Institute for Molecular Bioscience, University of Queensland, Brisbane, Queensland, Australia; 2 Watson School of Biological Sciences, Cold Spring Harbor Laboratory, Cold Spring Harbor, New York, United States of America; 3 Broad Institute, Cambridge, Massachusetts, United States of America; 4 MRC Functional Genomics Unit, Department of Physiology, Anatomy and Genetics, University of Oxford, Oxford, United Kingdom; 5 Department of Computer Science, University of Leipzig, Leipzig, Germany; 6 Department of Molecular and Experimental Medicine, Scripps Research Institute, La Jolla, California, United States of America; 7 Institut Curie, UMR3244-Pavillon Trouillet Rossignol, Paris, France; 8 Computational Biology and Bioinformatics, Yale University, New Haven, Connecticut, United States of America; 9 University of Miami, Miami, Florida, United States of America; 10 Omics Science Center, RIKEN Yokohama Institute, Tsurumi-ku, Yokohama, Kanagawa, Japan; University of California Berkeley, United States of America

## Abstract

Despite recent controversies, the evidence that the majority of the human genome is transcribed into RNA remains strong.

Current estimates indicate that only about 1.2% of the mammalian genome codes for amino acids in proteins. However, mounting evidence over the past decade has suggested that the vast majority of the genome is transcribed, well beyond the boundaries of known genes, a phenomenon known as pervasive transcription [1]. Challenging this view, an article published in *PLoS Biology* by van Bakel et al. concluded that “the genome is not as pervasively transcribed as previously reported” [2] and that the majority of the detected low-level transcription is due to technical artefacts and/or background biological noise. These conclusions attracted considerable publicity [3]–[6]. Here, we present an evaluation of the analysis and conclusions of van Bakel et al. compared to those of others and show that (1) the existence of pervasive transcription is supported by multiple independent techniques; (2) re-analysis of the van Bakel et al. tiling arrays shows that their results are atypical compared to those of ENCODE and lack independent validation; and (3) the RNA sequencing dataset used by van Bakel et al. suffered from insufficient sequencing depth and poor transcript assembly, compromising their ability to detect the less abundant transcripts outside of protein-coding genes. We conclude that the totality of the evidence strongly supports pervasive transcription of mammalian genomes, although the biological significance of many novel coding and noncoding transcripts remains to be explored.

## Previous Evidence for Pervasive Transcription

The conclusion that the mammalian genome is pervasively transcribed (i.e., “that the majority of its bases are associated with at least one primary transcript” [1]) was based on multiple lines of evidence. Both large-scale cDNA sequencing and hybridization to genome-wide tiling arrays were the major empirical sources of data. Analysis of full-length cDNAs from many tissues and developmental stages in mouse showed that at least 63% of the genome is transcribed and identified thousands of novel protein-coding transcripts and over 30,000 long noncoding intronic, intergenic, and antisense transcripts [7]–[9]. In parallel, whole chromosome tiling array interrogation of the RNA content of a variety of human tissues and cell lines revealed that, collectively, at least 93% of genomic bases are transcribed in one cell type or another [1],[10]–[13].

Since it is well established that highly expressed mRNAs dominate the non-ribosomal portion of the polyA+ transcriptome [7],[8],[10],[14]–[19], normalization approaches were used to reduce the quantity of highly expressed transcripts in these cDNA analyses [7],[8], and are implicit in tiling array approaches. This was necessary to allow the detection of rarer (often cell type–restricted [1],[13],[16],[19],[20]) transcripts.

The evidence for pervasive transcription also includes observations from a wide variety of other independent techniques (see reviews [21] and [22] for references). Indeed, a simple query of currently available human spliced EST data in GenBank shows that documented transcripts cover 57.09% of the genome. Because ESTs are largely generated from polyadenylated RNAs and do not exhaustively sample the transcriptome, this coverage represents the lower bound of genomic transcription.

Based on an analysis of genome-wide tiling arrays and short read RNA sequencing data, van Bakel et al. report that “most ‘dark matter’ transcripts (i.e., novel transcripts of unknown function) are associated with known genes” [2], a well-established and uncontroversial conclusion that has been reported previously [1],[8],[10],[13],[14],[19],[23]–[25] (see Text S1). Controversially, however, they also concluded that “the genome is not as pervasively transcribed as previously reported” [2]. The authors suggested that the discrepancy is explained by tiling arrays producing more false positive signals than previously appreciated, although they do not reconcile their conclusions with the extensive transcriptome cataloged by cDNA analyses [7],[8] and other approaches [21],[22]. The multi-centre ENCODE pilot project, for example, found that 74% of the bases in the genome areas analyzed were covered by primary transcripts identified by two independent technologies [1].

## Congruency of Tiling Array and Short Read RNA Sequencing Data

A major feature of van Bakel et al.'s argument was based on their comparison of precision recall (PR) curves generated from tiling arrays and RNA sequencing (RNA-seq), from which they concluded that tiling array results suffer from high false positive rates. These PR curves in principle measure the order in which transcribed regions are detected when the expression detection threshold is lowered in a stepwise manner. This analysis performed by van Bakel et al. indicated a large difference between transcribed regions detected by tiling arrays (referred to as transfrags or TARs since they are most often parts of longer transcripts) compared to those detected by RNA-seq (seqfrags). They showed that RNA-seq discovers known protein-coding exons at higher thresholds compared to unannotated transfrags, while tiling arrays found a larger fraction of non-exonic regions, even at high thresholds, from which they conclude a lower accuracy of tiling arrays.

There are two major limitations to this analysis (see also Text S1). First, the implication of lower accuracy of tiling arrays is made in the absence of an independent validation of the false positive rate (which, by contrast, was routinely conducted in previous tiling array studies using techniques such as RT-PCR, see e.g., [10],[13]). As explained later, correlating individual tiling array probes and RNA sequencing depth is not an appropriate comparison and cannot substitute as a validation method. Thus, the false-positive claim by van Bakel et al. is impossible to test precisely with the presented data.

Second, while RNA-seq offers linear quantification over a wide range, tiling arrays saturate at the upper end of signal strength. As a consequence, arrays are less reliable in distinguishing highly expressed known exons from less highly expressed novel transfrags, resulting in a lower precision value for any given recall in the PR curves. This fact explains much of the difference in shape between the curves, but does not imply that the regions detected by either technology are false positives, only that quantification by arrays is less linear than by RNA-seq, which is well understood.

**Figure 1:**
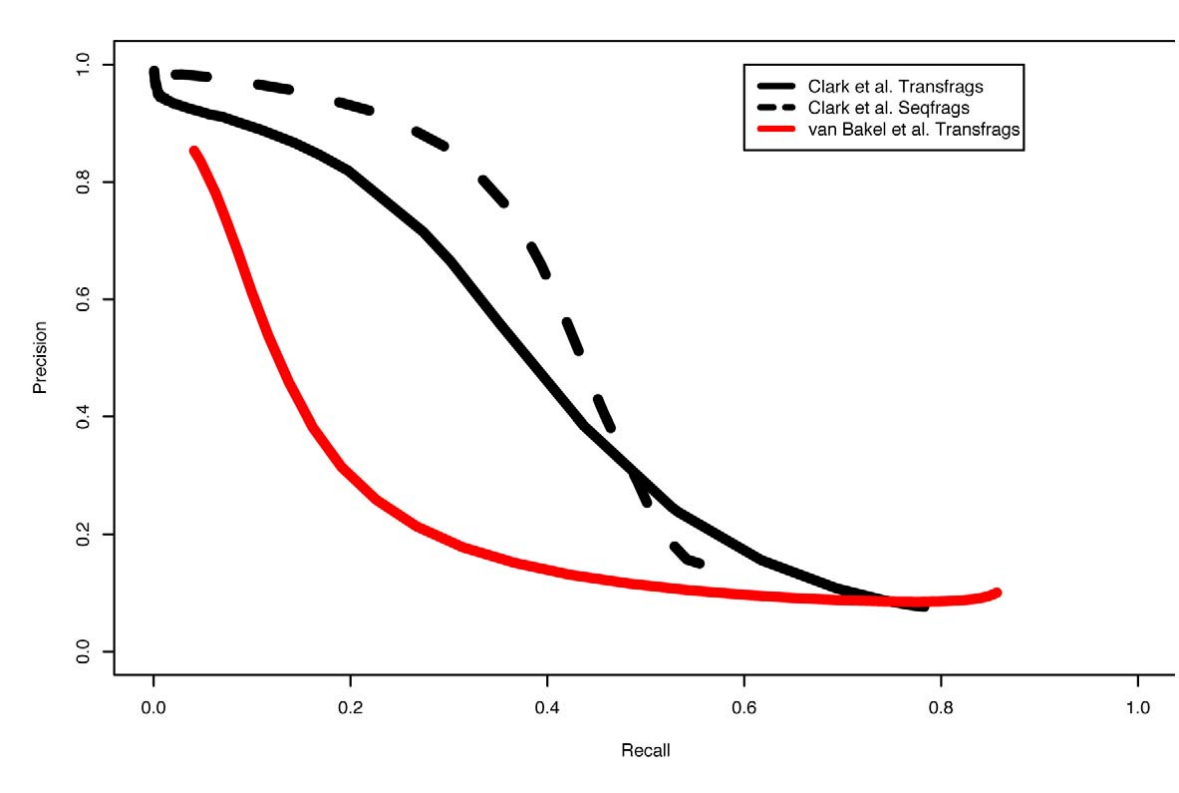
Precision recall curves for the overlap of transcribed regions (transfrags) detected in different experiments and annotated exons (from GENCODE version 4 and the UCSC known genes track from the UCSC Genome Browser). The Clark et al. transfrags are based on data generated as part of the ENCODE transcriptome project from K562 cell polyA+ RNA. The transfrags are generated from 5-bp two-color tiling arrays (MinRun  = 40 bp, MaxGap  = 40 bp). The seqfrags are based on 200 million paired-end 76 bp reads generated on the Illumina sequencing platform. Any detected region that overlaps an annotated exon is scored positive, all others negative. Fewer exons are detected overall (compared to van Bakel et al. [2]), likely reflecting the difference between a relatively homogeneous cell line and complex tissue like brain.

We performed a similar PR curve analysis using ENCODE tiling array data for K562 cellular RNA, and found results that are substantially different from those reported by van Bakel et al., but consistent with a recent analysis of a sample-matched nematode RNA-seq and tiling array dataset by Agarwal et al. (2010) [26]. Briefly, we identified transfrags on the tiling arrays with a range of different thresholds. Similar to the analysis by van Bakel et al., every transfrag that overlapped any annotated exon was scored as positive, while all others were scored negative. The resulting PR curve was dramatically different from the curve presented in Figure 1A of van Bakel et al. Moreover, the shape of the PR curve and the precision for any given recall level for our tiling arrays is much closer to the van Bakel et al. sequencing data and to our own sequencing data from a matched K562 sample (Figures 1 and S1). These results suggest that, while decreased dynamic range of tiling arrays leads to an increased number of non-exonic regions being detected at high thresholds (lower initial precision values; see Figure 1), the difference between sequencing and tiling arrays is not large and the discrepancies identified by van Bakel et al. appear to be specific to their analysis.

A second argument for lower accuracy of tiling arrays by van Bakel et al. was based on the observation that there is a relatively low correlation between individual probe-level signals from arrays and sequencing read depth. Unfortunately, such a statement reveals a fundamental lack of understanding of tiling array technology. Tiling arrays are neither intended nor designed to give reliable signals from each individual probe. The more appropriate analysis for correlation is at the level of transcribed regions such as exons or transfrags, as has been done previously [26] and which generally shows a good correlation. This also points to another problem in the van Bakel et al. study, which used tiling arrays with 36-bp spaced probes, offering only very few probes for each exon. The 5-bp spaced (7X resolution) arrays used for ENCODE (and in many published human transcriptome studies) offer more power to filter individual probe-level noise (Figures S2, S3, and S4). Overall it appears that, while RNA-seq offers better linearity of quantification and much higher resolution for boundaries of transcribed regions, the overall detection accuracy of tiling arrays is not significantly lower. This is also in agreement with the recent analysis by Agarwal et al. [26], which consistently observed intergenic and intronic transcription.

Finally, it is difficult to reconcile the purported high false positive nature of the tiling array results with numerous previous studies that validated up to 94% identified transcripts using independent techniques such as RT-PCR, RACE, and Northern blot analyses [10]–[13],[27].

## Detection and Interpretation of Low-Level Transcription

We suggest that the overarching conclusions drawn by van Bakel et al.—that there is only spasmodic (not pervasive) low-level transcription of much of the genome, and that much of this transcription has “random character” [2]—are the result of a number of debatable aspects of their logic and analysis. These may be summarized as (1) insufficient sequencing depth and breadth and poor transcript assembly, together with the sampling problems that arise as a consequence of the domination of sequence data by highly expressed transcripts; compounded by (2) the dismissal of transcripts derived from introns; (3) a lack of consideration of non-polyadenylated transcripts; (4) an inability to discriminate antisense transcripts; and (5) the questionable assertion that rarer RNAs are not genuine and/or functional transcripts.

1. *Sequencing depth, breadth, and assembly.* The conclusions of van Bakel et al. about the pervasiveness of transcription were based on transcript read number, not the extent of genomic coverage of the observed transcripts (which is the correct metric), stating “the vast majority of sequence reads in polyA+ samples correspond to known genes and transcripts, arguing against widespread transcription to the extent reported previously”. The former fact does not justify the consequential argument. This also highlights a key caveat of RNA sequencing—i.e., diminishing returns—whereby abundant transcripts constitute the majority of reads, making rare transcripts difficult to find in the absence of normalization approaches. This problem is clearly evidenced in the van Bakel et al. dataset, where ∼88% of unique polyA+ sequences mapped to exons of known genes, which comprise just over 2% of the genome. Therefore, the transcription in the remainder of the genome was sampled by only ∼12% of the reads.

This insufficient depth of sequencing is illustrated by comparing the rates of discovery for exonic, intronic, and intergenic sequences as sequencing depth increases. Despite continuing to constitute most reads, the area covered by exons quickly moves towards saturation, while the area covered by intronic and intergenic transcripts was found to “keep increasing at roughly constant rates” [2]. Thus, the sequence coverage of the vast majority of the genome is not saturated, and potentially includes many novel protein-coding and noncoding transcripts insufficiently sampled at the given read depth.

Underscoring the importance of adequate transcriptome sampling, concurrently published deep sequencing studies, with two to three times greater depth of data from polyA+ RNAs from cultured cells, were still not saturating [16],[28]. Nonetheless, and unsurprisingly, the increased sequencing depth led to increased novel transcript discovery, as only 70% of the identified splice junctions were derived from “known genes” in a mouse myoblast cell line [28], compared to 94% reported by van Bakel et al. Re-analysis of transcript assembly at different sequencing depths also suggested, crucially, poor assembly and poor recovery of lowly expressed transcripts at the deepest level of sequencing used by van Bakel et al. [28].

The lack of sequencing depth in the van Bakel et al. study was exacerbated by the pooling of 10 tissues/cell lines and the use of such a highly complex tissue as brain. Increasing the complexity of the sample dilutes the relative proportion of tissue- and cell type–specific transcripts. Using the brain (170 billion cells) [29], we calculate that a cell type–specific transcript present at ∼10 copies per cell (a common level of abundance) in 0.1% of cells would have only a ∼50% chance of being detected by any reads at the depth of sequencing utilized by van Bakel et al., let alone of being assembled into a complete transcript (see Text S1).

**Figure 2:**
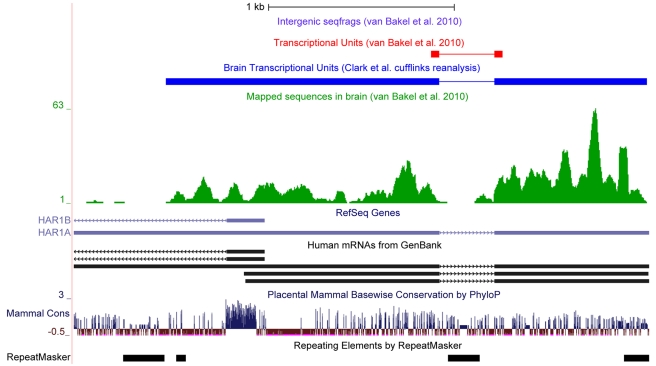
Poor coverage of single intron transcriptional units (TUs) by van Bakel et al. [**2**]. The figure shows the abundance of sequence reads mapped to the *HAR1A* locus [43] (green) and the TU created from these tags by van Bakel et al. using TopHat (red) [44]. In contrast, the Clark et al. TU created by reanalysis of sequence reads using Cufflinks [28] includes the extended 5′ and 3′ exonic sequences (dark blue).

Importantly, because the genomic strand from which individual sequence reads were derived was unknown in their study, the method that van Bakel et al. employed to assemble these reads into transcriptional units required that contigs in the vicinity of known genes be bounded by splice sites or cross a splice site, automatically excluding (i) nearly all 5′ and 3′ UTRs; (ii) deep sequencing reads, other than the splice site, in genes with a single intron (Figure 2); (iii) transcripts from single exon genes, such as the highly expressed metastasis associated lung adenocarcinoma transcript 1 (*MALAT1*) (Figure S5), and transcripts (not containing a splice site) originating from introns [30]; and (iv) perhaps most importantly, any known transcript for which there was no identifiable splice junction in the dataset. This methodology therefore discriminates against lowly expressed transcripts, heavily biasing in favor of common mRNAs.

2. *Intronic transcripts.* Despite the data showing that 51.4% of the genomic area covered by their human reads aligns with intronic regions, van Bakel et al. presumptively dismissed these sequences as mainly derived from unprocessed pre-mRNAs, due to their “low coverage and ubiquitous character”. Intronic regions, which correspond to more than a third of the genome, are by definition transcribed, and hence must be included in estimates of the amount of transcription across the genome. It is also important to note that many introns are not fixed entities and whether a genomic region is intronic, intergenic, or exonic depends on the cell type and physiological state of the cell. In addition, the number of functional RNAs that may be derived from introns is unknown, although there is considerable evidence that they can produce a diversity of discrete stable RNA products from both the sense and antisense strands [12],[15],[31],[32]–[34], including novel RNAs with validated functions (e.g., [35]).

3. *Non-polyadenylated RNAs.* The data used by van Bakel et al. to support the conclusion that “dark matter transcripts make up a small fraction of the total sequenced transcript mass” focused on polyadenylated RNA. However, previous transcriptomic analyses showed that over 40% of non-ribosomal transcripts are non-polyadenylated [13], and more recent deep sequencing of total RNA has revealed that over 45% of uniquely mapping sequence reads originate from intronic and intergenic regions [36],[37], compared to only 10% in the polyA+ RNA from equivalent samples examined by van Bakel et al.

4. *Antisense and overlapping transcription.* Tiling array, cDNA, EST, and RNA sequencing evidence all indicate that considerable interleaved transcription occurs on both strands [1],[9],[23],[36], with at least 66% of all protein-coding genes in mouse showing evidence of overlapping or antisense transcription [9]. However, van Bakel et al. concluded that their data “argue against widespread interleaved transcription of protein-coding genes”. This discrepancy can be explained in large part by the lack of strand information in the RNA sequencing data used by them to assemble transcriptional units (TUs). Indeed, the assembled TUs covered less than 26% of the genome (compared to over 40% spanned by RefSeq genes) and, tellingly, less than 2% of RefSeq annotated 3′UTR sequences. This lack of coverage and strand information resulted in a large underestimate of the extent of antisense and overlapping transcription (Figures S6 and S7), for which functional evidence is also emerging (see e.g., [38]).

5. *Discriminating low signal strength from background noise.* The assertion of van Bakel et al. that low sequence coverage (by seqfrags) equates with transcriptional “by-products” and/or “random initiation events” is highly debatable. Such seqfrags might equally, if not more, plausibly reflect stochastic sampling of transcripts that are less expressed, less stable, and more cell specific [39]. This is not proof or even evidence of irrelevance. Moreover, van Bakel et al. infer non-functionality of rare transcripts without any biological data, but one cannot expect vast numbers of novel coding and noncoding RNAs to be functionally annotated coincident with their discovery, especially if, as is likely, they have many different functions [40]. The yeast *GAL10*-ncRNA provides a good example: despite a steady-state expression level of around one transcript per 14 cells, it is functional [41]. Similarly, the mammalian HOTTIP RNA plays an important role in epigenetic regulation despite an average expression level of around 0.3 transcripts per cell in expressing tissues [42]. Therefore, while expression levels are important, it cannot be assumed a priori that low expression equates to non-functionality.

## Summary

A close examination of the issues and conclusions raised by van Bakel et al. reveals the need for several corrections. First, their results are atypical and generate PR curves that are not observed with other reported tiling array data sets. Second, characterization of the transcriptomes of specific cell/tissue types using limited sampling approaches results in a limited and skewed view of the complexity of the transcriptome. Third, any estimate of the pervasiveness of transcription requires inclusion of all data sources, and less than exhaustive analyses can only provide lower bounds for transcriptional complexity. Although van Bakel et al. did not venture an estimate of the proportion of the genome expressed as primary transcripts, we agree with them that “given sufficient sequencing depth the whole genome may appear as transcripts” [2].

There is already a wide and rapidly expanding body of literature demonstrating intricate and dynamic transcript expression patterns, evolutionary conservation of promoters, transcript sequences and splice sites, and functional roles of “dark matter” transcripts [39]. In any case, the fact that their expression can be detected by independent techniques demonstrates their existence and the reality of the pervasive transcription of the genome.

## Supporting Information

Text S1 Supplementary text.
(0.22 MB DOC)

Figure S1 Comparison of the PR curve transfrags from Clark et al. (ENCODE) and van Bakel et al. data.
(0.27 MB PDF)

Figure S2 Histogram of transfrag length for van Bakel et al. [1] and Clark et al. (ENCODE) transfrags.
(0.26 MB PDF)

Figure S3 PR curve for transcripts.
(0.23 MB PDF)

Figure S4 Genome browser screenshots showing annotation and transfrags from the van Bakel et al. and the ENCODE tiling arrays using a threshold that gives similar recall values for both.
(0.14 MB PDF)

Figure S5 Known single exon transcripts are missing from van Bakel et al. TUs. Sequence reads (green) provide good coverage of *Malat1* gene but are not found in the van Bakel et al. TUs (red).
(0.19 MB PDF)

Figure S6 Lack of UTR coverage in TUs prevents the detection of overlapping transcripts.
(0.22 MB PDF)

Figure S7 Lack of UTR coverage in TUs prevents the detection of chains of overlapping transcripts.
(0.27 MB PDF)

## Abbreviations

PR: precision recall
