# Supplementary material for: The Reality of Pervasive Transcription
Source: PLoS Biol. 2011 Jul 12;9(7):e1000625. doi: 10.1371/journal.pbio.1000625 (PMC3134446; doi:10.1371/journal.pbio.1000625)
Supplement: Text S1 — Supplementary text. (0.22 MB DOC) [file pbio.1000625.s001.doc]

**Text S1**

**Genomic distribution of ‘dark matter’ transcripts**

The headline of van Bakel *et al.* that ‘most “dark matter” transcripts are associated with known genes’ [1] is a well-established and uncontroversial conclusion that has been reported in previous studies [2-10]. Indeed, not only do ‘known genes’ (including their introns) encompass a large fraction of the genome, but many independent studies and technologies have demonstrated that most transcription is concentrated around these genes, leading Kapranov *et al.* [9] to propose “a model of genome organization where protein-coding genes are at the center of a complex network of overlapping sense and antisense (long) RNA transcription”.

**Other limitations of PR curve analysis**

The small changes in the shape of the curves in Figure 1B of van Bakel *et al*. do not show that the sequencing depth of the presented RNA-seq libraries is sufficient and hence are not good evidence for a high false positive rate from tiling arrays. Increasing the read depth of RNA-seq is not expected to change the order in which transfrags are discovered and hence should not greatly affect the shape of the PR curve, since for any given number of detected exons (recall value) the number of novel transfrags with similar expression levels does not change. Nevertheless, deeper libraries will discover more true novel transfrags at any fixed read count threshold, consistent with the prior findings that novel transcripts are on average less highly expressed than annotated exons. This trend is indeed observed in Figure 2C of van Bakel *et al.* [1].

**The problem with pooling**

Pooling of complex tissues severely limits detection of low-level cell type specific transcripts. Mammalian cells contain an estimated 400 000 mRNAs (not including a similar number of nuclear and or polyA- RNAs) with expression levels that vary over 4 orders of magnitude [11-13]. Much of the mRNAs population is made up of a relatively small number of moderately and highly expressed genes, with the bulk of genes more lowly expressed at between 1 and 30 transcript copies per cell [11-14]. Studies of the transcriptome have also demonstrated a high degree of tissue specific expression between cell types and tissues, especially among novel and lowly expressed transcripts [3,4,8,15]. For example, 40% of all transfrags (representing both known and novel transcripts) were found to be cell line specific in the ENCODE pilot project [8].

The number of cell types in the brain is very large, with ~60 different neuronal cell types in retina, while the CA1 region of the hippocampus (the simplest of all the cortices) has around two dozen types of inhibitory interneurons alone, without counting other classes of neurons [16]. Given the large number of brain regions a fairly conservative extrapolation of this data suggests at least 1000 neuronal cell-types in the brain (plus non-neuronal brain cell subtypes) [16,17].

How then would sequencing from a complex tissue such a brain compromise the ability to detect mRNAs expressed at this low (but common) level in a cell type specific manner?

A transcript present at a (low but common) level of 10 RNAs per cell, present in 0.1% of brain cells (~170 million cells [18]) would be diluted to 1 transcript per 40 million in a whole brain sample. Assuming an equal detection probability for each RNA, a library depth of 40 million reads would be necessary to detect just one read from this transcript. Assuming this mRNA is of average length (~2kb) and given the paired-end sequencing of ~200mers carried out by van Bakel *et al*. [1], a sequencing depth of 400 million reads would be needed to completely cover this transcript. The sequencing carried out by van Bakel *et al*. in the brain was to a depth of ~23 million paired-end reads of which less than 20 million could be mapped to the genome, therefore the van Bakel *et al.* dataset would have at best a 50% chance of detecting a single read from a cell-type specific transcript expressed at an average level.

However the problem is even worse, as neurons only comprise at most 50% of cells in the brain and only ~20% of cells in the cerebral cortex [18], so a gene specific to a cortical layer subtype could reasonably be found in 0.02% of brain cells, requiring 200 million reads to detect and 2 billion reads to cover this transcript.

Furthermore, it is clear that many transcripts are expressed at less than 10 copies/cell [11-13]. A 2kb transcript found in 0.1% of brain cells with an average expression level of 1copy/cell would require 400 million reads to be detected and 4 billion to be covered. This is well below the detection level of RNA sequenced from whole brains, with less than 1 in 10 transcripts expressed at this level detected at all in the van Bakel *et al*. dataset [1]. Given these results it is not surprising that lowly expressed reads look “random”, as they have been stochastically sampled from a population of rare RNAs.

These calculations also show that requiring a sequencing depth two or more orders of magnitude deeper than carried out by van Bakel *et al*. to fully characterize these sequences does not suggest “these transcripts may largely be attributed to biological and/or technical background”. Instead it reflects the effects of pooling samples of high biological complexity.

**References**

1. van Bakel H, Nislow C, Blencowe BJ, Hughes TR (2010) Most "dark matter" transcripts are associated with known genes. PLoS Biol 8: e1000371.

2. Kapranov P, Cawley SE, Drenkow J, Bekiranov S, Strausberg RL, et al. (2002) Large-scale transcriptional activity in chromosomes 21 and 22. Science 296: 916-919.

3. Kampa D, Cheng J, Kapranov P, Yamanaka M, Brubaker S, et al. (2004) Novel RNAs identified from an in-depth analysis of the transcriptome of human chromosomes 21 and 22. Genome Res 14: 331-342.

4. Cheng J, Kapranov P, Drenkow J, Dike S, Brubaker S, et al. (2005) Transcriptional maps of 10 human chromosomes at 5-nucleotide resolution. Science 308: 1149-1154.

5. Carninci P, Kasukawa T, Katayama S, Gough J, Frith MC, et al. (2005) The transcriptional landscape of the mammalian genome. Science 309: 1559-1563.

6. Engstrom PG, Suzuki H, Ninomiya N, Akalin A, Sessa L, et al. (2006) Complex Loci in human and mouse genomes. PLoS Genet 2: e47.

7. Khaitovich P, Kelso J, Franz H, Visagie J, Giger T, et al. (2006) Functionality of intergenic transcription: an evolutionary comparison. PLoS Genet 2: e171.

8. Birney E, Stamatoyannopoulos JA, Dutta A, Guigo R, Gingeras TR, et al. (2007) Identification and analysis of functional elements in 1% of the human genome by the ENCODE pilot project. Nature 447: 799-816.

9. Kapranov P, Cheng J, Dike S, Nix DA, Duttagupta R, et al. (2007) RNA maps reveal new RNA classes and a possible function for pervasive transcription. Science 316: 1484-1488.

10. Fejes-Toth K, Sotirova V, Sachidanandam R, Assaf G, Hannon GJ, et al. (2009) Post-transcriptional processing generates a diversity of 5'-modified long and short RNAs. Nature 457: 1028-1032.

11. Jackson DA, Pombo A, Iborra F (2000) The balance sheet for transcription: an analysis of nuclear RNA metabolism in mammalian cells. FASEB J 14: 242-254.

12. Bishop JO, Morton JG, Rosbash M, Richardson M (1974) Three abundance classes in HeLa cell messenger RNA. Nature 250: 199-204.

13. Soares MB, Bonaldo MF, Jelene P, Su L, Lawton L, et al. (1994) Construction and characterization of a normalized cDNA library. Proc Natl Acad Sci U S A 91: 9228-9232.

14. Hastie ND, Bishop JO (1976) The expression of three abundance classes of messenger RNA in mouse tissues. Cell 9: 761-774.

15. Carninci P, Waki K, Shiraki T, Konno H, Shibata K, et al. (2003) Targeting a complex transcriptome: the construction of the mouse full-length cDNA encyclopedia. Genome Res 13: 1273-1289.

16. Stevens CF (1998) Neuronal diversity: too many cell types for comfort? Curr Biol 8: R708-710.

17. Nelson SB, Hempel C, Sugino K (2006) Probing the transcriptome of neuronal cell types. Curr Opin Neurobiol 16: 571-576.

18. Azevedo FA, Carvalho LR, Grinberg LT, Farfel JM, Ferretti RE, et al. (2009) Equal numbers of neuronal and nonneuronal cells make the human brain an isometrically scaled-up primate brain. J Comp Neurol 513: 532-541.
