# Supplementary material for: The Reality of Pervasive Transcription
Source: PLoS Biol. 2011 Jul 12;9(7):e1000625. doi: 10.1371/journal.pbio.1000625 (PMC3134446; doi:10.1371/journal.pbio.1000625)
Supplement: Figure S1 — Comparison of the PR curve transfrags from Clark et al. (ENCODE) and van Bakel et al. data. (0.27 MB PDF) [file pbio.1000625.s002.pdf]

Figure S1

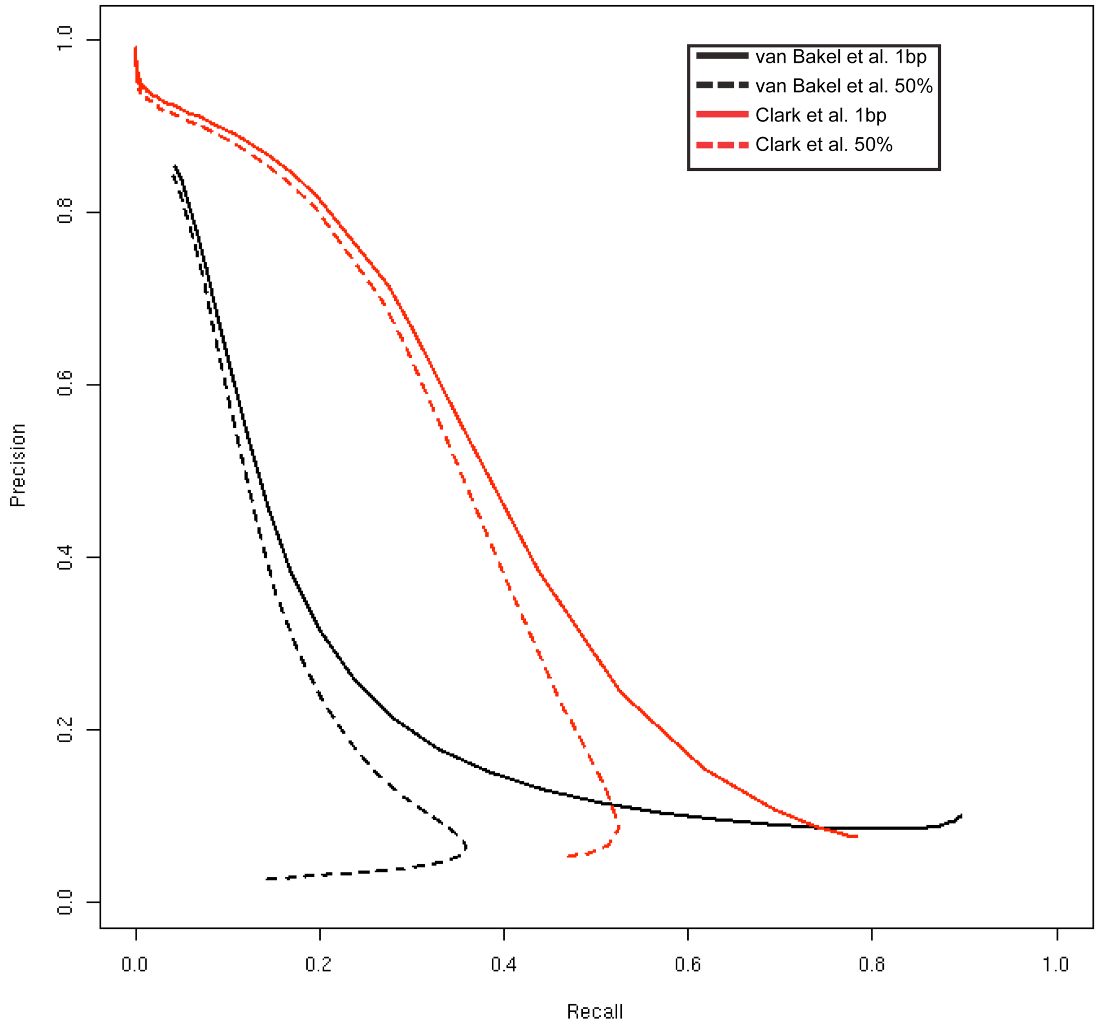

Figure S1: Comparison of the PR curve transfrags from Clark *et al.* (ENCODE) and van Bakel *et al.* data [1]. The dramatic difference in shape holds even for strict overlap criteria, requiring that any transfrag overlaps an exon with at least 50% of its length to prevent random overlaps. At very low probe thresholds (1 bp overlap), transfrags increase in length resulting in fewer positive calls under the 50% overlap rule.

1. van Bakel H, Nislow C, Blencowe BJ, Hughes TR (2010) Most "dark matter" transcripts are associated with known genes. PLoS Biol 8: e1000371.
