# Supplementary material for: The Reality of Pervasive Transcription
Source: PLoS Biol. 2011 Jul 12;9(7):e1000625. doi: 10.1371/journal.pbio.1000625 (PMC3134446; doi:10.1371/journal.pbio.1000625)
Supplement: Figure S2 — Histogram of transfrag length for van Bakel et al. [1] and Clark et al. (ENCODE) transfrags. (0.26 MB PDF) [file pbio.1000625.s003.pdf]

Figure S2

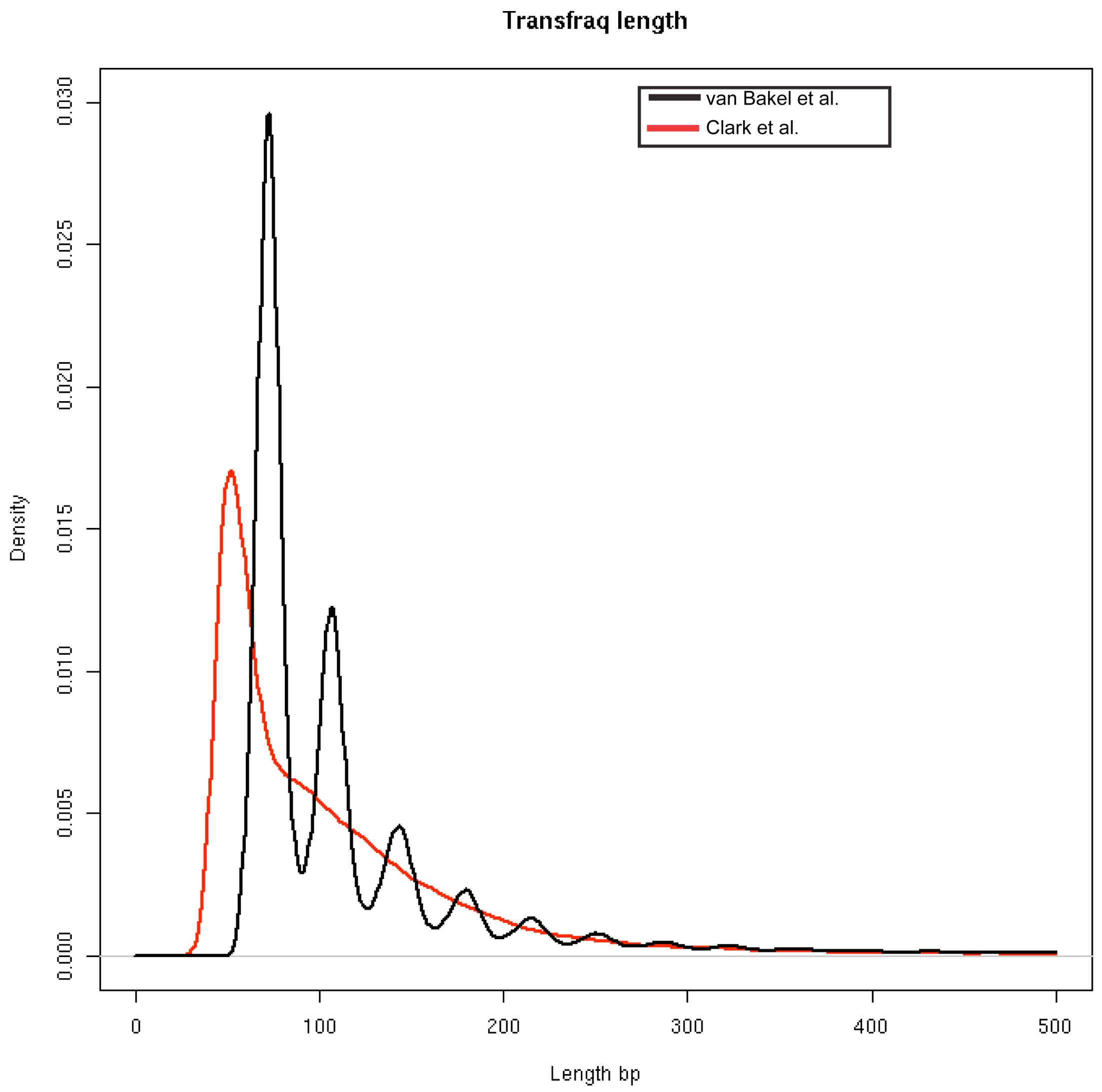

Figure S2: Histogram of transfrag length for van Bakel et al. [1] and Clark *et al.* (ENCODE) transfrags. The average length of transfrags in the two datasets is comparable. The discrete peaks in the van Bakel et al. data result from the 36bp spacing of probes.

1. van Bakel H, Nislow C, Blencowe BJ, Hughes TR (2010) Most "dark matter" transcripts are associated with known genes. PLoS Biol 8: e1000371.
