# Supplementary material for: The Reality of Pervasive Transcription
Source: PLoS Biol. 2011 Jul 12;9(7):e1000625. doi: 10.1371/journal.pbio.1000625 (PMC3134446; doi:10.1371/journal.pbio.1000625)
Supplement: Figure S3 — PR curve for transcripts. (0.23 MB PDF) [file pbio.1000625.s004.pdf]

Figure S3

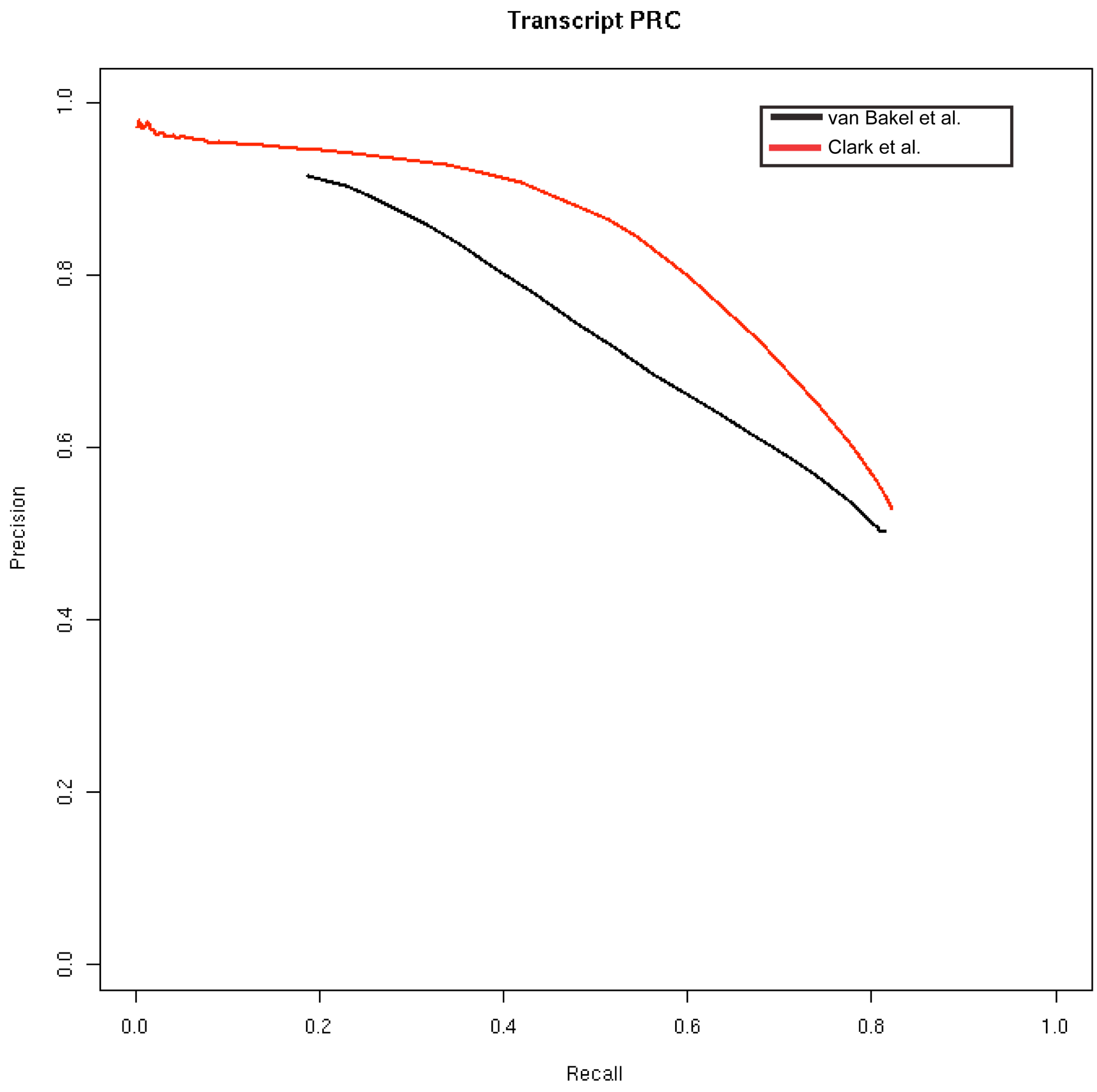

Figure S3: PR curve for transcripts. If any transfrag that overlaps any part of a transcript (as opposed to only the exons) is scored positive, the van Bakel *et al.* dataset [1] shows much higher precision, indicating that many of their negative calls (transfrags detected outside of exons) from Figure 1 result from transfrags in introns. A similar analysis for the Clark *et al.* data is shown for comparison.

1. van Bakel H, Nislow C, Blencowe BJ, Hughes TR (2010) Most "dark matter" transcripts are associated with known genes. PLoS Biol 8: e1000371.
