# Supplementary material for: The Reality of Pervasive Transcription
Source: PLoS Biol. 2011 Jul 12;9(7):e1000625. doi: 10.1371/journal.pbio.1000625 (PMC3134446; doi:10.1371/journal.pbio.1000625)
Supplement: Figure S4 — Genome browser screenshots showing annotation and transfrags from the van Bakel et al. and the ENCODE tiling arrays using a threshold that gives similar recall values for both. (0.14 MB PDF) [file pbio.1000625.s005.pdf]

Figure S4

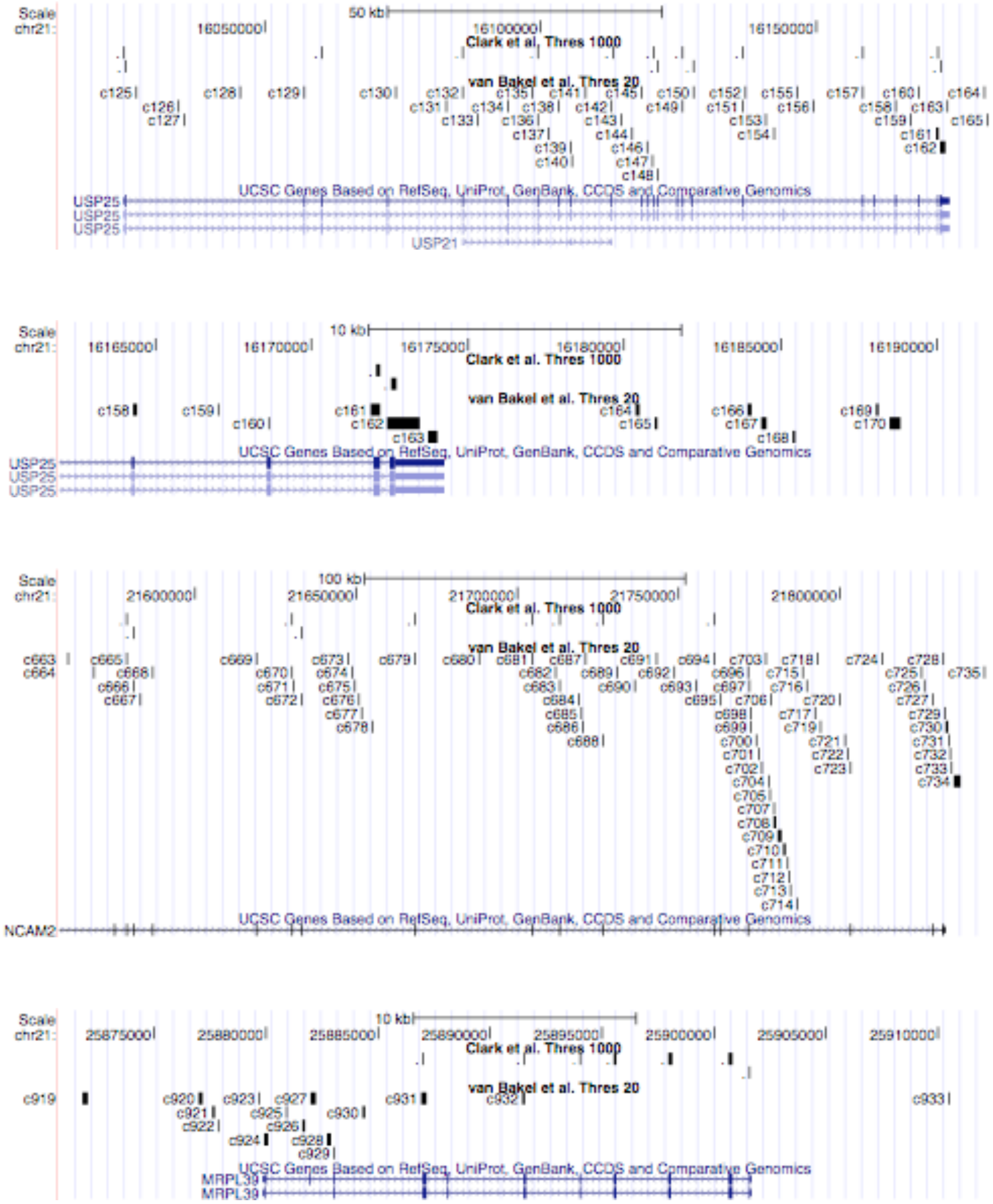

Figure S4: Genome browser screenshots showing annotation and transfrags from the van Bakel *et al*. [1] and the Clark *et a*l. (ENCODE) tiling arrays using a threshold that gives similar recall values for both. The van Bakel *et al*. arrays show many more scattered transfrags.

1. van Bakel H, Nislow C, Blencowe BJ, Hughes TR (2010) Most "dark matter" transcripts are associated with known genes. PLoS Biol 8: e1000371.
