# Supplementary material for: The Reality of Pervasive Transcription
Source: PLoS Biol. 2011 Jul 12;9(7):e1000625. doi: 10.1371/journal.pbio.1000625 (PMC3134446; doi:10.1371/journal.pbio.1000625)
Supplement: Figure S5 — Known single exon transcripts are missing from van Bakel et al. TUs. Sequence reads (green) provide good coverage of Malat1 gene but are not found in the van Bakel et al. TUs (red). (0.19 MB PDF) [file pbio.1000625.s006.pdf]

Figure S5

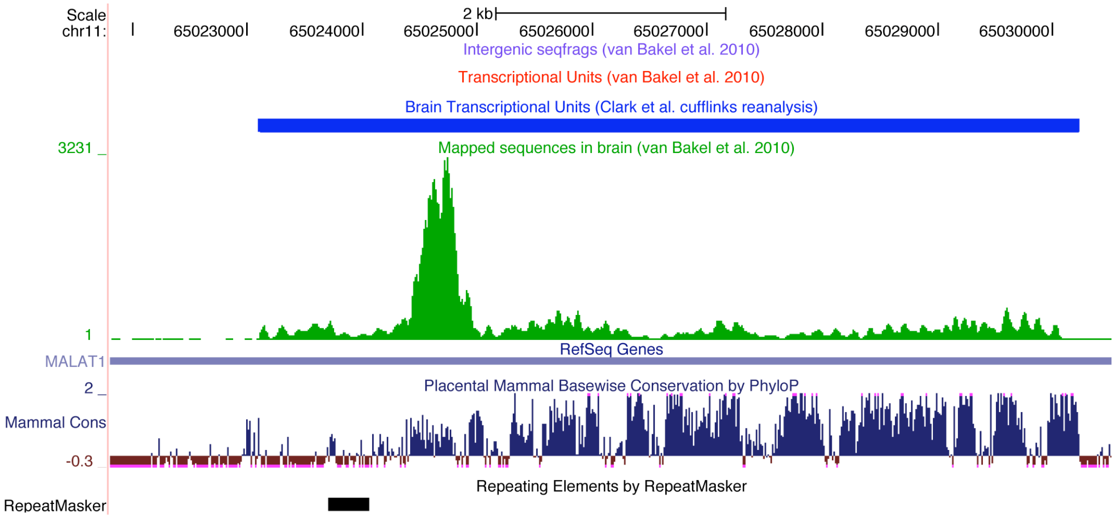

Figure S5: Known single exon transcripts are missing from van Bakel *et al.* TUs. Sequence reads (green) provide good coverage of *Malat1* gene but are not found in the van Bakel *et al*. TUs (red). Reanalysis of sequence reads without specifying the presence of a splice junction in known genes identifies the relatively highly expressed *Malat1* transcript [1] as a TU (dark blue).

1. Ji P, Diederichs S, Wang W, Boing S, Metzger R, et al. (2003) MALAT-1, a novel noncoding RNA, and thymosin beta4 predict metastasis and survival in early-stage non-small cell lung cancer. Oncogene 22: 8031-8041.
