# Supplementary material for: The Reality of Pervasive Transcription
Source: PLoS Biol. 2011 Jul 12;9(7):e1000625. doi: 10.1371/journal.pbio.1000625 (PMC3134446; doi:10.1371/journal.pbio.1000625)
Supplement: Figure S6 — Lack of UTR coverage in TUs prevents the detection of overlapping transcripts. (0.22 MB PDF) [file pbio.1000625.s007.pdf]

# Figure S6

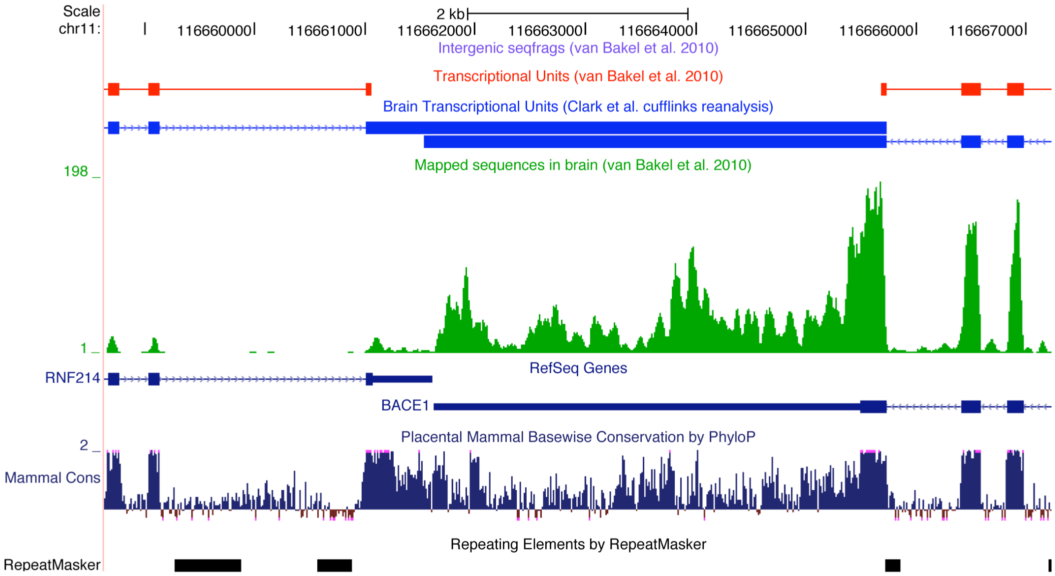


Figure S6: Lack of UTR coverage in transcriptional units prevents the detection of overlapping transcripts. Sequence reads (green) provide evidence for overlap of the relatively highly expressed *BACE1* gene [1] and the more lowly expressed *RNF214*. Exclusion of 3'UTR sequence in the TUs created by van Bakel *et al*. [2] prevents detection of this overlap, which is identified by including 3'UTR sequence (dark blue). Only representative cufflinks transcripts are shown.

1. Vassar R, Kovacs DM, Yan R, Wong PC (2009) The beta-secretase enzyme BACE in health and Alzheimer's disease: regulation, cell biology, function, and therapeutic potential. J Neurosci 29: 12787-12794.

2. van Bakel H, Nislow C, Blencowe BJ, Hughes TR (2010) Most "dark matter" transcripts are associated with known genes. PLoS Biol 8: e1000371.
