# Supplementary material for: The Reality of Pervasive Transcription
Source: PLoS Biol. 2011 Jul 12;9(7):e1000625. doi: 10.1371/journal.pbio.1000625 (PMC3134446; doi:10.1371/journal.pbio.1000625)
Supplement: Figure S7 — Lack of UTR coverage in TUs prevents the detection of chains of overlapping transcripts. (0.27 MB PDF) [file pbio.1000625.s008.pdf]

Figure S7

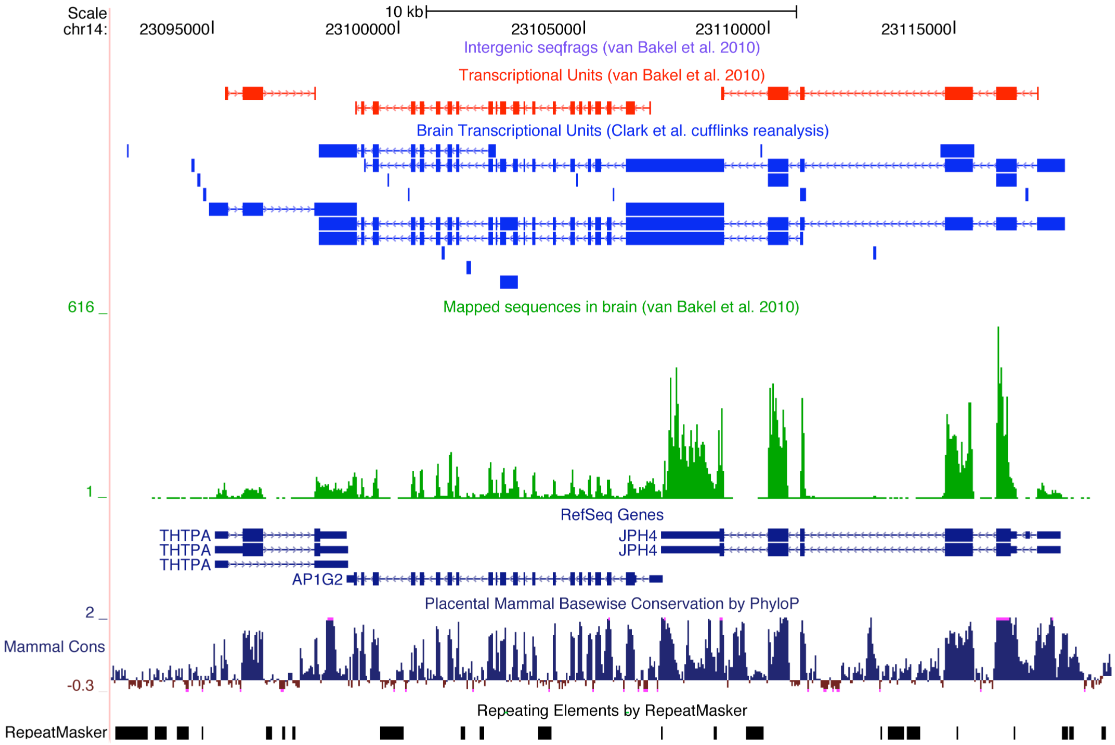

Figure S7: Lack of UTR coverage in transcriptional units prevents the detection of chains of overlapping transcripts. Sequence reads (green) provide evidence for transcription through this complex locus, which is confirmed by our reanalysis (dark blue) showing the presence of many overlapping transcripts, some of which are likely incomplete due to lack of sequence depth. The lack of UTRs and unspliced transcripts in the TUs created by van Bakel *et al*. [1] prevented the detection of this transcriptional overlap and complexity.

1. van Bakel H, Nislow C, Blencowe BJ, Hughes TR (2010) Most "dark matter" transcripts are associated with known genes. PLoS Biol 8: e1000371.
